# Genomic and Bioinformatics Analysis of Familial Partial Lipodystrophy Type 3 Identified in a Patient with Novel PPARγ Mutation and Robust Response to Pioglitazone

**DOI:** 10.3390/ijms252212060

**Published:** 2024-11-10

**Authors:** Abdulrahman Hummadi, Saeed Yafei, Dhayf Alrahman Mutawwam, Raed Abutaleb, Yahia Solan, Abdullah Khawaji, Ali Jaber Alhagawy, Turki Algohani, Mamdouh Khardali, Mohammed Hakami, Abdulrraheem Daghriri, Wegdan Hezam, Nourah Kariri

**Affiliations:** 1 Adult Endocrinology and Diabetes, Jazan Endocrinology & Diabetes Center, Ministry of Health, Jazan 82723, Saudi Arabia; 2 Endocrinology Department, Faculty of Medicine and Health Sciences, Taiz University, Taiz 11712, Yemen; 3 Molecular Genetics Department, Jazan Health Affairs, Ministry of Health, Jazan 82617, Saudi Arabia; 4 Pharmacology Department, Jazan Endocrinology & Diabetes Center, Ministry of Health, Jazan 82723, Saudi Arabia; 5 Altewal Hospital, Ministry of Health, Jazan 82723, Saudi Arabia

**Keywords:** lipodystrophy, familial partial lipodystrophy, FPLD, Pioglitazone, PPARγ

## Abstract

Familial partial lipodystrophies (FPLDs) are very rare inherited disorders characterized by partial loss of adipose tissue from the upper and lower extremities. At least seven subtypes of FPLD have been identified and are mostly dominantly inherited. FPLD type 3 is caused by mutations in the PPARγ gene, which encodes for the protein peroxisome proliferator-activated receptor gamma (PPARγ). We identified a Saudi female with PFLD3 presented with partial lipoatrophy, uncontrolled diabetes, severe hypertriglyceridemia, and recurrent pancreatitis. The clinical and biochemical findings in this proband were described before and after treatment with Pioglitazone in addition to the conventional treatment. DNA extraction and whole exome sequencing (WES) were performed to detect the variant. The mutant gene was subjected to Sanger analysis to confirm the results. We applied five specific computational prediction tools to assess the pathogenicity of variation, namely the MT, DANN, CADD, BayesDel, and fitCons tools. We assessed protein modeling and stability with the AlphaFold-generated structures for both wild-type and mutant proteins. Finally, we conducted molecular docking using the AutoDock Vina virtual docking. Upon whole exome sequencing, a *c.1024C>T p.(Gln342Ter)* missense mutation was detected in the PPARγ gene associated with FPLD3. This variant is a novel mutation that has not been described in all genome databases. Sanger analysis confirmed the heterogenicity and pathogenicity of this variant. All five computational prediction tools indicate that this variant is considered highly pathogenic. Our patient showed a dramatic response to Pioglitazone, a synthetic PPARγ agonist. From structural modeling, we found that the enhanced binding affinity of the mutant PPARγ protein to Pioglitazone likely improves the activation of PPARγ, enhancing its transcriptional activity and resulting in better clinical outcomes. These findings extend the spectrum of PPARγ mutations responsible for FPLD3 and highlight the potential for personalized treatment strategies based on genetic mutations.

## 1. Introduction

Lipodystrophy syndromes are rare heterogeneous disorders that can be inherited or acquired. They are characterized by selective partial or generalized loss of adipose tissue with subsequent development of insulin resistance and other metabolic complications [1]. The two main subtypes of genetic lipodystrophies are congenital generalized lipodystrophy (CGL) and familial partial lipodystrophy (FPLD) [2].

Familial partial lipodystrophies (FPLDs) are inherited mostly as autosomal dominant and rarely as autosomal recessive disorders [3]. At least seven different subtypes of FPLD have been identified [4]. FPLD type 3 (FPLD3) is an autosomal dominant lipoatrophy caused by mutations in the peroxisome proliferator-activated receptor gamma (PPARγ) gene, located on the short arm of chromosome 3 [5]. The PPARγ gene encodes for the PPARγ protein, a nuclear receptor expressed in adipose tissues, responsible for adipocyte differentiation and regulation of insulin sensitivity [2,6]. PPARγ mutations may inhibit adipocyte differentiation, leading to peripheral subcutaneous fat loss from the arms and legs with preservation of fat in the truncal region, neck, and face [2,6]. Similar to other forms of lipodystrophies, FPLD3 is characterized by a cluster of metabolic disorders including insulin resistance, diabetes mellitus, early-onset hypertension, hypertriglyceridemia, lipotoxicity, low-grade inflammation, altered adipokine secretion, and ectopic fat tissue accumulation [7].

Worldwide, the prevalence of FPLD is very rare, with an estimated prevalence of 1.3–4.7 cases per million [8], and only about 60 patients with FPLD3 have been reported [4]. In Saudi Arabia, the prevalence of lipodystrophies is unknown. We thought that the prevalence of lipodystrophies would be high due to the high frequency of consanguineous marriages [9]. Only few cases of congenital generalized lipodystrophy were reported previously, of which two sisters with CGL were reported from our center [10]. However, no similar cases with FPLD were reported in Saudi Arabia, which may be due to underestimation or non-publication. We, herein, report the clinical and molecular characteristics of the first case of FPLD3 with novel PPARγ mutation.

## 2. Results

### 2.1. Whole Exome Sequencing and Variant Filtrations

The patient had approximately 120,000 variations (our CADD Phred quality score parameter was Q37). Our strict standard for variable filtration depended on the genomic datasets of the global population, such as the 1K Genomes database and gnomAD, with an allele frequency of less than 1% (MAF < 0.01) among various ethnic groupings. We included all variations within the exons and splice sites of the coding genomic region. Another variant that corresponded to both upstream and downstream, intergenic intronic, 5′ and 3 regions of the gene—as well as synonymous variants—were not included.

We identified one heterozygous stop-gain variant in the PPARγ gene among the filtered results for the genes associated with familial partial lipodystrophies. This variant is NM_015869.4 c.1024C>T p.(Gln342Ter), on 3p25.2 exon 4, which is located within chromosome 3 at position 12,416,908 (Supplementary Table S1). The variant has no reference SNP ID or reported allele frequency in the dbSNP, 1k Genomes, gnomAD, or any of the other genome databases. Also, when we applied computational prediction tools such as MT, DANN, CADD, BayesDel, and fitCons, the results indicated that this variant is a highly pathogenic variant.

### 2.2. Sanger Sequencing

**Figure 1:**
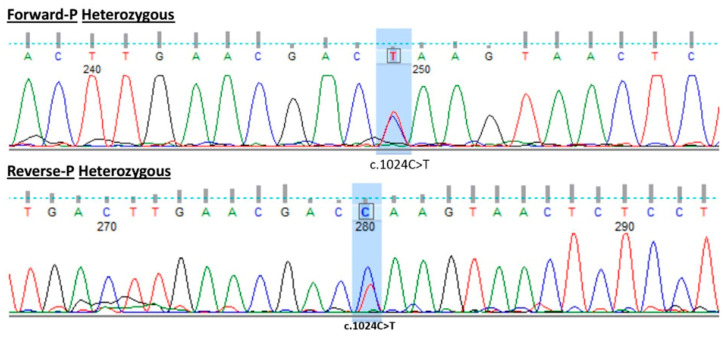
Forward and reverse Sanger sequencing result. The chromatogram shows Sanger sequencing results for the PPARγ gene, with the forward and reverse strands presented in a heterozygous state. The peaks are color-coded: green for Adenine (A), red for Thymine (T), blue for Cytosine (C), and black for Guanine (G), for clear nucleotide identification. Blue boxes highlight the c.1024C>T mutation in both strands, while dotted lines align nucleotide positions for accurate sequence reference.

The PPARγ gene mutation was verified by Sanger sequencing to detect modification, segregation, and heterozygosity in this variant. Sanger sequencing analysis confirmed that the most likely causing factor that led to FPLD3 in this patient is the presence of the heterozygous c.1024C>T p.(Gln342Ter) variant of the PPARγ gene. This missense variant results in stop codon termination of glutamine at amino acid position 342 p.(Gln342Ter). Both forward and reverse primer sequences were used to verify the heterozygous character of the mutation, therefore confirming the pathogenicity of this variant (Figure 1). FPLD3 is an autosomal dominant disease; however, in this case, the parents were not available for segregation analysis to confirm the mode of inheritance.

### 2.3. Comprehensive Analysis of PPARγ Gene Variants

The PPARγ gene located within chromosome 3 at position (12,416,858–12,416,958) is very important for metabolism, regulating fat storage, and has a significant role in glucose metabolism, adiposeness, and inflammation response [11]. This gene contains multi-transcript variants, including different protein-coding transcripts (such as PPARγ-201, PPARγ-202, PPARγ-204), retained introns (PPARγ-225, PPARγ-223), and nonsense-mediated decay (PPARγ-221, PPARγ-207). The gnomAD database contains both structural variants (SVs) and various short variants (Indels and SNPs) within this gene, which indicate a wide range of mutations such as synonymous, missense, stop-gain, and frameshift mutations. The PPARγ contains highly constrained and conserved elements through 91 mammals, confirming their evolutionary importance. The information taken from Ensembl version 112.38 (GRCh38.p14) on Homo Sapiens provides a comprehensive view of the PPARγ gene, its transcript, and related genetic variations, which provides a reliable and insightful vision for its functional mechanisms and potential roles for many phenotypes and diseases.

### 2.4. Prediction Analysis

**Figure 2:**
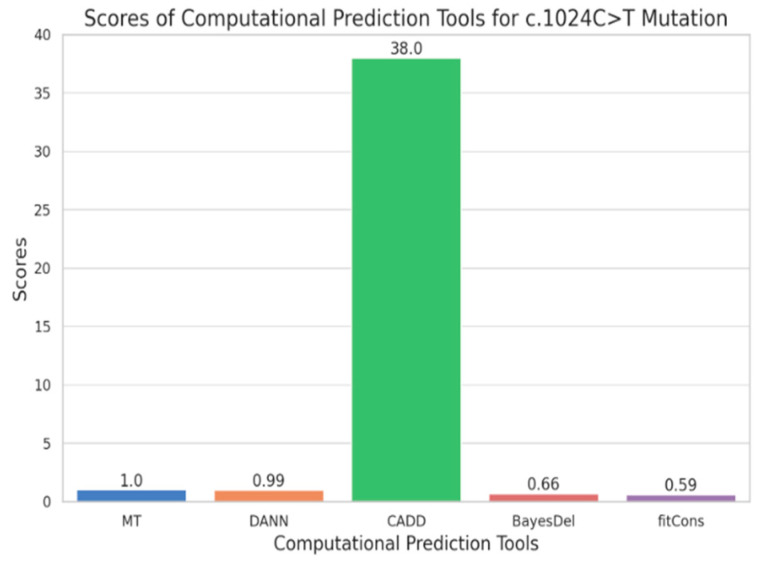
Predicting scores of the deleteriousness of genetic variants.

We implemented five computational prediction tools, MT, DANN, CADD, BayesDel, and fitCons, that help in determining the effect of the c.1024C>T mutation on protein function. MT is used to assess the potentiality to which genetic mutation may lead to disease. DANN is a tool based on deep learning and is designed to predict the deleteriousness of genetic mutations. CADD is a tool that incorporates multiple annotations into a single metric to predict the score of deleteriousness of genetic variants. BayesDel is a Bayesian-based tool that combines various types of evidence for predicting the deleteriousness of genetic variants. MT, DANN, CADD, and BayesDel prediction scores of the c.1024C>T variant were 0.1, 0.1, ≥20, 0.1, and 0.1, respectively (Figure 2). These results indicate that it is a deleterious mutation. According to the Franklin ACMG classification [12], this variant is classified as pathogenic based on the PVS1, PM2 category (Supplementary Figure S2). The PVS1 criteria pertain to very strong pathogenic missense variants. This variant has no allelic frequency, while PM2 criteria pertain to extremely low frequency in gnomAD population databases, so it has not been reported in the Uniport and ClinVar databases.

The statistical analysis revealed a mean score of about 8.25 with a standard deviation of 16.63, which indicates significant pathogenic variation among the tools. The ANOVA shows statistically significant differences in value (statistic = 51,964.49, *p*-value < 0.001). Furthermore, a correlation matrix showed a perfect correlation (1.0) among the scores, which indicates that while each tool employs a different scoring system, their outputs collectively contribute to assessing the pathogenicity of variants. A bar graph showing the scores assigned by each tool will further highlight CADD’s dominance in predicting the deleteriousness of the c.1024C>T variant, underscoring the importance of integrating multiple computational tools in assessing the effect of mutation.

### 2.5. Conserved Analysis

**Figure 3:**
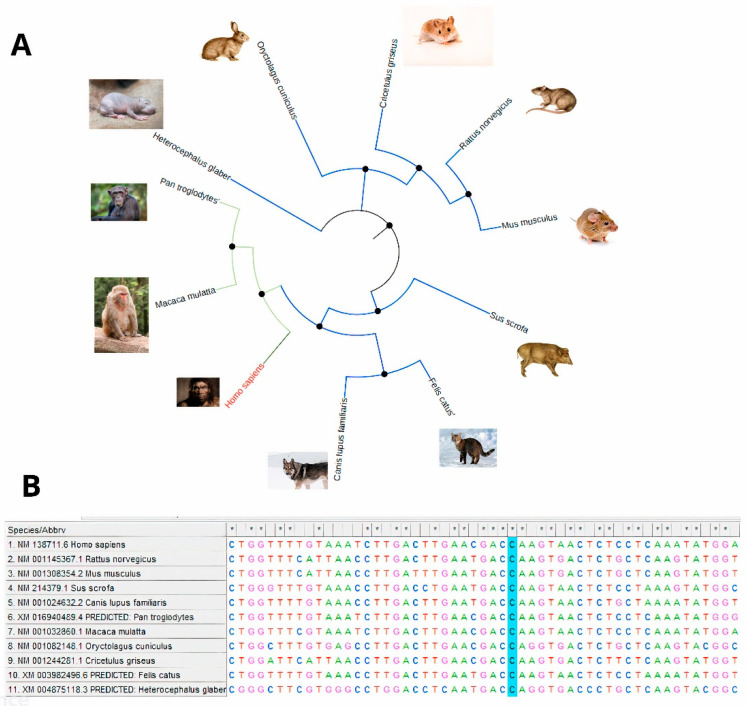
(**A**) Phylogenetic tree of the PPARγ gene. (**B**) Nucleotide sequence alignment of human and primate PPARγ genes. The asterisk marks a conserved region across different species, while the blue background indicates the identified mutation. Different colors highlight the nucleotides: Adenine (A), Thymine (T), Cytosine (C), and Guanine (G) for clarity.

In our study, we built a phylogenetic tree to demonstrate the evolutionary relationships between different species for the PPARG gene sequences. The tree was constructed using sequences from eleven species, including humans and other primate mammals such as Pan troglodytes, Rattus norvegicus, Sus scrofa, and Canis lupus familiaris (Supplementary Table S2). These sequences were aligned to evaluate the conservation of the c.1024C>T variation across different primates. The phylogeny demonstrates that this genomic region is highly conserved across diverse species, which indicates the significance of this mutation. Furthermore, the multiple sequence alignment highlights the presence of conserved nucleotides surrounding the c.1024 position, emphasizing its potential functional importance in evolutionary terms. The sequences used for both the phylogenetic tree and alignment were obtained from the NCBI database, with their corresponding accession numbers provided in (Figure 3).

### 2.6. Molecular Docking Between PPARγ and Pioglitazone

**Figure 4:**
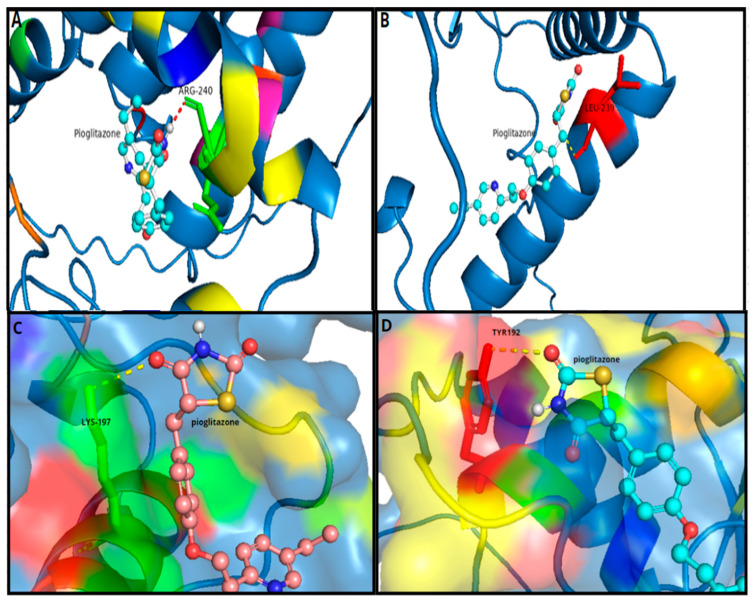
PPARγ and Pioglitazone docking. (**A**,**C**) are wild-type binding sites. (**B**,**D**) are mutant binding sites.

**Figure 5:**
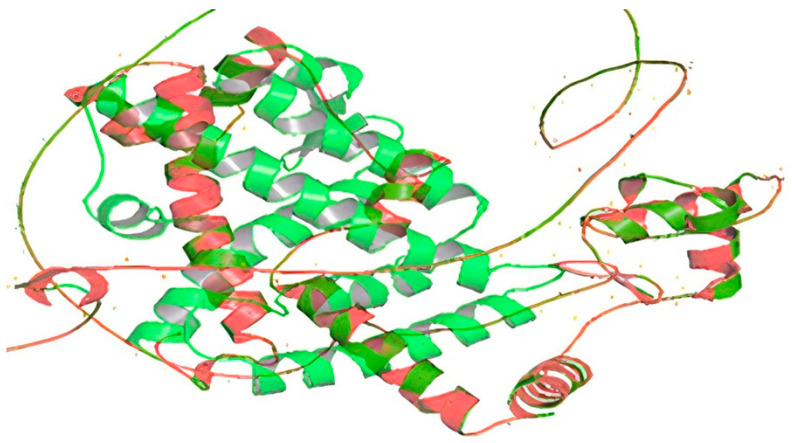
Comparison structure between the wild-type (green) and mutant (red) forms of the PPARγ gene. The green color represents the native conformation, while the red highlights structural changes caused by the mutation.

Through using the AutoDock Vina program [13], we docked the PPARγ protein (wild-type and mutant) with the Pioglitazone ligand, and we found two unique binding sites associated with the mutant form. The docking binding affinity of the Pioglitazone ligand complex and wild-type PPARγ was −5.854 and −5.416 (kcal/mol), whereas the Pioglitazone ligand complex and mutant PPARγ protein was −5.957 and −6.026 (kcal/mol). LYS-197 (Lysine) and ARG-240 (Arginine) are the wild-type residues we found that interact with Pioglitazone. Conversely, mutants form interaction residues with TYR-192 (Tyrosine) and LEU-239 (Leucine) (Figure 4 and Figure 5).

The increased binding affinity and efficacy of Pioglitazone in the mutant form may be attributed to the structural alteration and different interaction residues. The root mean square deviation (RMSD) score of the docked position of the mutant and wild-type forms is 0.054 Å. This result indicated that the overall 3D structure of the protein was not appreciably changed by the mutation. Molecular docking shows that the binding energy for the mutant-Pioglitazone complex is lower than that of the wild-type-Pioglitazone complex of the PPARγ protein. This implies that the mutant protein binds Pioglitazone more strongly than the wild-type protein. As lower binding energy usually equates to increased binding affinity, the Pioglitazone has bound to c.1024C>T p. (Gln342Ter) quite successfully.

## 3. Discussion

We described a case of FPLD3 in a Saudi female presenting with lipoatrophic diabetes, insulin resistance, and hypertriglyceridemia caused by a novel heterozygous pathogenic missense variant of c.1024C>T mutation in the PPARγ gene. This missense variant results in stop codon termination of glutamine at amino acid position 342 p.(Gln342Ter), NM_015869.4, which is a new variant associated with autosomal dominant FPLD3. Such a variant has not been reported previously for FPLD3, so it broadens the spectrum of genetic factors causing FPLD3.

The mutation of the c.1024C>T within the PPARγ gene occurs in a highly conserved region across human and different primate species, as demonstrated in Figure 3. The conservation of a sequence, especially one conserved across evolutionarily distant species, generally implies that it has a critical biological function and is likely under strong evolutionary pressure to remain unchanged. In the case of PPARγ, the protein plays a key role in regulating lipid metabolism, insulin sensitivity, and adipocyte differentiation. Therefore, any alteration in such a highly conserved region is likely to compromise these functions. The c.1024C>T mutation brings about the change in glutamine (Gln) residue to a premature stop codon, which finally produces a truncated nonfunctional protein in the context of the protein’s structure and role.

Based on the results of prediction analysis, the c.1024C>T is a deleterious mutation and is strongly predicted to be pathogenic according to the ACMG standards [12]. However, the biological effect of this mutation needs further studies.

FPLD3 is a rare autosomal dominant genetic disorder that was first described by Barroso et al. in 1999 [5]. In previous studies, most patients with FPLD3 were females of different ages who typically presented after the onset of puberty [14]. The phenotypic features vary in the reported cases, but gluteal and lower extremity clinical lipoatrophy with preservation of fat in the face and trunk was common in most, as it was in our case [15,16].

Patients with FPLD3 usually have loss-of-function mutations in the PPARγ gene [14]. The PPARγ nuclear receptor is highly expressed in adipose tissues and encodes by the PPARγ gene. PPARγ is a key factor in adipocyte differentiation, lipogenesis, lipolysis, lipid uptake, and storage [6]. It is suspected that mutated PPARγ inhibits adipocyte differentiation, so the fatty tissue loses its ability to correctly synthesize and store triglycerides to free fatty acids and glycerol from stored triglycerides [17]. Previous studies reported that a reduction in PPARγ protein’s transcriptional activity of ≥ 30% is sufficient to cause partial lipodystrophy [14]. The PPARγ protein is composed of four functional domains, of which the most essential are DBD (DNA-binding domain) and LBD (ligand-binding domain). Mutations in either DBD or LBD protein domains have been reported to cause FPLD3, but no significant difference in the clinical phenotype between mutations located in different domains [15]. One explanation postulated that the amount of reduction in the transcriptional activity of the PPARγ protein, rather than the location of the mutation, could determine the clinical phenotype [15].

Metabolic complications of FPLD3 depend on the level of reduction in PPARγ expression and the extent of lipoatrophy [17]. Patients with extensive lipoatrophy have severe metabolic complications such as DM, hypertension, hypertriglyceridemia, hirsutism, polycystic ovaries, hepatic steatosis, hypertriglyceridemia, and hypercholesterolemia [4]. All of these features were confirmed in our case, except for hypertension, as she has been normotensive till now. Moreover, masculinization, hirsutism, polycystic ovaries, and hyperandrogenism in our case were also documented in other females with FPLD3 [14].

Therapeutic interventions for patients with lipoatrophic diabetes and FPLD are limited. Current options include strict fat and calorie restriction, metformin, insulin, and glucagon-like peptide 1 agonists. Metreleptin has been tried in some cases with very low leptin levels [8]. Thiazolidinediones, a class of synthetic high-affinity PPARγ agonists, have been used in FPLD3, leading to improved insulin sensitivity and adipogenesis with variable response [18].

We started our patient on a controlled dietary program, adjusting insulin doses, and started 15 mg of Pioglitazone daily, increasing the dose to 30 mg thereafter in addition to fenofibrate 145 mg, long-chain omega-3 fatty acids, and metformin 1500 mg per day. Within three months of treatment, we found dramatic improvements in glycemic control and hypertriglyceridemia. After three years of the current regimen, her HbA1c is at 6.7%, her insulin doses have decreased from a total dose of 198 units per day to about 90 units per day, and her triglyceride level is below 250 mg in different visits. There has been no recent admission due to pancreatitis, and her BMI is maintained between 27.4 kg/m^2^ to 28.7 kg/m^2^. Her lipoatrophy is mildly improved, with moderate improvement in hirsutism and acanthosis nigricans. Agostini et al. [18] reported that Pioglitazone 30 mg/day in a female with FPLD3 resulted in dramatic and sustained improvements in glycemic control and dyslipidemia. They also reported improvement in hirsutism, hyperandrogenism, and acanthosis nigricans. Iizaka, T. et al. [19] reported that Pioglitazone leads to an increase in adiponectin level, improvement in insulin resistance, and mild increase in subcutaneous fat without a change in leptin levels. However, adiponectin and leptin levels in our case did not change significantly. The clinical efficacy of Pioglitazone in our patient could not be explained by the decrease in insulin resistance alone, but might be related to molecular interaction between Pioglitazone and the mutant PPARγ gene, at least in this case of a c.1024C>T variant.

Thiazolidinediones, such as Pioglitazone, are synthetic PPARγ ligands that have been licensed for the treatment of type 2 diabetes. These synthetic ligands alter the transcription of genes influencing carbohydrate and lipid metabolism, resulting in changed amounts of protein synthesis. Pioglitazone improves glycemic control in people with T2D by improving insulin sensitivity through its action at PPARγ and affects lipid metabolism through its action at PPARα. Pioglitazone had a good safety profile with some reported side effects. The most common side effects of Pioglitazone include weight gain, pedal edema, osteoporosis, and precipitation of heart failure in at-risk populations without any increase in overall cardiovascular mortality. Pioglitazone was also linked to bladder cancer in some reports, so the use of this medication is restricted in elderly patients and patients with hematuria or those with a high risk of bladder cancer.

The use of Pioglitazone for the treatment of FPLD3 has mixed results [18]. A hypothesis by Agostini et al. [18] said that patients with FPLD3 might be resistant, show the usual response, or be hyperresponsive to Pioglitazone and other TZD compounds. This different response depends mainly on the nature of the mutation and the mutation ligand complex interaction. In our case, the structural comparison between the wild-type and mutant forms of the PPARγ gene, with an RMSD of 0.054 Å over 335 atoms, indicates minimal deviation, suggesting the mutation does not significantly alter the protein’s overall 3D conformation. However, this patient with the mutant PPARγ gene shows a markedly high response to Pioglitazone. This enhanced response may result from subtle changes in the protein’s active or allosteric sites, improving drug binding or efficacy. Clinically, this mutation likely affects the receptor’s interaction with Pioglitazone or its downstream signaling pathways, leading to improved therapeutic outcomes.

The docking results indicate that the mutant PPARγ protein binds to Pioglitazone with higher affinity compared with the wild-type, as shown by the lower binding energy, but the biological significance of this increased affinity needs more elaboration to understand its potential impact on clinical presentation. In this context, the mutations in PPARγ of FPLD 3 may lead to a decrease in normal protein function, which affects lipid storage and glucose metabolism. The enhanced binding affinity between the mutant protein and Pioglitazone suggests that Pioglitazone may stabilize or restore partial functionality of the mutant PPARγ, potentially leading to improved regulation of glucose and lipid homeostasis in this patient. This could be translated clinically to better metabolic control, reduced insulin resistance, and improved overall management of lipodystrophy symptoms in patients carrying the mutation. Further experimental validation is necessary to link these docking findings with tangible therapeutic outcomes, but the higher binding affinity hints at a more effective molecular interaction, which may explain the patient’s positive response to Pioglitazone.

We performed five computational prediction tools—DANN, MT, CADD, BayesDel, and fitCons—to evaluate the impact of the c.1024C>T mutation on protein function. The results we obtained from MT and DANN indicate a strong likelihood of the mutation being deleterious, reinforcing their reliability in predicting pathogenicity. Of all those tools, CADD is the most significant due to its comprehensive approach, integrating various annotations to improve the accuracy of the predictions. This tool’s output significantly supports the classification of the mutation as pathogenic, given its high score that exceeds conventional thresholds for pathogenicity. On the other hand, fitCons and BayesDel offer indications of possible harmful effects, though with a moderate level of assurance. In combination, these tools align in their predictions of the variant’s harmful nature, underscoring the importance of using multiple computational methods for a robust assessment of genetic mutations.

The unique characteristics of LEU-239 and TYR-192 in the mutant PPARγ protein contribute strongly to the strong and efficient binding of Pioglitazone. The hydroxyl group in TYR-192 forms hydrogen bonds with the acceptor hydrogen bond in Pioglitazone. Additionally, the aromatic ring engages in π-π stacking, which highly interacts with the aromatic rings of Pioglitazone, allowing for strong and specific binding. Additionally, the pi stacking (π-π stacking) interactions occur between aromatic rings, where π-electrons in the rings attract each other without forming a direct chemical bond. In this case, the aromatic ring of the protein engages in π-π stacking with the aromatic rings of pioglitazone. This interaction enhances the binding strength and specificity between the protein and pioglitazone, contributing to a stable and effective drug-protein complex.

Also, TYR-192′s polar nature facilitates interactions with polar functional groups in the drug. LEU-239 amino acid, characterized by its unique hydrophobic side chain, interacts with the hydrophobic parts of Pioglitazone, depending on van der Waals forces, contributing to the stability of the drug-protein complex. The aromatic ring, Pioglitazone, and the thiazolidinedione ring participate in π-π stacking interactions and hydrogen bonding, respectively, while non-polar residues such as LEU-239 interact with hydrophobic regions. These complementary binding sites where TYR-192 provides polar and π-π stacking interactions and LEU-239 contributes hydrophobic interactions create a strong and stable binding environment for Pioglitazone. This enhanced binding affinity likely improves the activation of PPARγ, enhancing its transcriptional activity and resulting in better clinical outcomes for the patient. In summary, we reported a rare case of FPLD3 caused by a new PPARγ mutation, c.1024C>T, with a dramatic response to Pioglitazone.

## 4. Materials and Methods

### 4.1. Case History

The proband was a 32-year-old Saudi female referred to our endocrinology clinic with a more than 13-year history of uncontrolled diabetes mellitus and hypertriglyceridemia. Menarche started at the age of 12 years, her menstruation is scanty with oligomenorrhea, and she was diagnosed with polycystic ovary syndrome. She developed diabetes mellitus at the age of 17 and her diabetes was almost uncontrolled, despite the high dose of basal-bolus insulin regimen, with a total daily dose of 198 units/day. Her medical records reported very high triglyceride levels that reached 2430 mg/dL at one visit and which decreased gradually thereafter. Her history did not reveal ketoacidosis, despite continuous high blood sugar, and she was admitted twice for episodes of acute pancreatitis. Family history revealed a consanguineous relationship with her parents, she had no sisters or brothers, and her father died ten years ago with acute abdomen after a long history of diabetes.

On examination, her body mass index (BMI) was 28.5 kg/m^2^, blood pressure within normal range, cushingoid face, with a truncal increase of fat. Her body habitus appears masculine female with hypertrophy of the calf muscle and upper limbs and eruptive xanthomas in the abdomen and back. Hirsutism was clear on the face, chin, chest, and upper and lower limbs. She had partial fat loss on her lower extremities and buttocks, while the subcutaneous fat was preserved in her face, abdomen, and trunk.

Her laboratory findings on presentation are shown in Table 1. Her fasting serum glucose was 352 mg/dL, HbA1c was >14%, triglycerides and total cholesterol were mildly elevated, and serum alanine aminotransferase was higher than normal. Investigations also included normal fasting c-peptide, high insulin level, normal thyroid stimulating hormone, negative glutamic acid decarboxylase antibody, and normal fasting leptin and adiponectin levels. Abdominal ultrasonography revealed multiple small ovarian cysts and hepatic steatosis. Gathering this information together, the patient achieved the diagnostic criteria for FPLD [2], and a sample of venous blood was withdrawn for genetic study to confirm the diagnosis. Before taking part, the patient signed informed consent, and a clinical geneticist looked over her medical records. Family history revealed that she is her parents’ only daughter, with no siblings, her father died of diabetes, and her mother was non-diabetic and refused to give consent for genetic analysis. Her pedigree is shown in Supplementary Figure S1.

Current management includes nutritional and exercise programs in addition to metformin, multiple daily insulin injections, fenofibrate, and long-chain omega-3 fatty acids. Pioglitazone 15 mg/day was added, and the dose was increased to 30 mg daily, considering the presence of insulin resistance. On subsequent visits, her glycemic measures and triglyceride levels improved. After three years of treatment, her HbA1c is within the target range of 6.7%, while lipoatrophy mildly improves.

### 4.2. Molecular Genetic Tests

#### 4.2.1. DNA Extraction

Through using the best manufacturing practices and techniques of the Haven Scientific DNAbler–Blood Kit, we used white blood cell samples to extract deoxyribonucleic acid (DNA). By using 1.0% agarose gel electrophoresis, the DNA was run to record the high molecular weight DNA to pass the whole exome sequencing (WES) exome library generation QC process. DNA purity and concentration were calculated using high-performance liquid chromatography (HPLC).

#### 4.2.2. WES Analysis

We extracted 200 ng/L genomic DNA from the affected patient and used it to build exome libraries using the Roche NimbleGen SeqCap EZ Exome Kit. An An Illumina NovaSeq 6000 Illumina, Inc., San Diego, CA, USA was used to generate high-throughput short-read sequencing. Variants were found by using SAM and GATK tools under the reads passed strict sequence QC Phred value (>30). After that, the data from the WES were filtered by quality, pathogenicity, inheritance patterns, and minor allele frequencies (<1%). Using reference databases including GME Variome Project, ExAC, gnomAD, 1000 Genomes Project [20], and All of Us (https://databrowser.researchallofus.org (accessed on 16 March 2024)), all allelic frequencies of selected variants were verified [21].

#### 4.2.3. Sanger Sequencing for Segregation and Validation Variant

The FPLD mutation found by whole exome sequencing (WES) was subjected to Sanger analysis to confirm the result. We built oligonucleotide primers encompassing a region of at least 120 base pairs on either side of the mutation through the applied Primer-BLAST program from NCBI at https://www.ncbi.nlm.nih.gov/tools/primer-blast (accessed on 16 March 2024). Strike requirements included a guanine-cytosine (GC) ratio over 40%, a melting point above 50 degrees Celsius, and a primer length of approximately 20 base pairs, with consideration to prevent hairpin formation and self-annealing. Then, we used BioEdit software (version 7.7.1, released on 10 May 2021) for sequence alignment with the reference gene of the candidate gene and to identify the position and properties of this variant [22].

### 4.3. Computational Analysis

#### 4.3.1. Prediction Tools

The variant effect predictor (VEP) tool from the Ensembl server was used to predict pathogenicity for possible variants. Pathogenicity of variation was assessed using specific tools such as MT, DANN, CADD, BayesDel, and fitCons according to their prediction scores. For this evaluation, we entered variation information, such as the nucleotide sequence location and mRNA reference sequence, using (VEP) to get scores and then analyze the results. Finally, we use the Franklin website, which assists in variant classification based on ACMG recommendations. This analysis comprised data from functional analysis, de novo occurrences, allelic data, segregation patterns, and computational analysis of current population research.

#### 4.3.2. Comparative Genomics and Phylogenetic Analysis

Conserved sequence analysis plays a crucial role in understanding the extent to which the mutation site is conserved across generations through many distinct species that have the same nucleotide sequence. We used the sequence conservation tool from the Ensembl website to analyze the variant position of the evolutionary conserved regions in ten primates. After that, we apply a phylogenetic tree by using the target gene to illustrate the evolutionary links between other primates and humans and detect if our mutation is present within a highly conserved region or not.

#### 4.3.3. Protein Modelling and Stability

We used AlphaFold-generated structures, which produce very reliable results. The PDBsum online server [23] was used for both wild-type and mutant proteins by uploading their amino acid. This method makes the secondary structures analysis of loops, β-pleated sheets, and α-helices fast, easy, and reliable. After that, we used DUET to investigate the effects of amino acid changes on protein stability. This method uses SVMs, mCSM, and SDM to predict the stability strength of a protein.

#### 4.3.4. Molecular Docking

We used AutoDock Vina virtual docking [24], which aims to enhance and develop the graphic interaction system. Our procedure started with the target protein being configured and bioactive compounds in the format of a PDBQT file. Utilizing AutoDock Vina’s ‘Maximize’ function, the physical dimensions of the docking grid box surrounding the ligand were ascertained to ensure that, during docking, protein surface regions are available and reachable. The box’s real measurements are as follows: First ligands are box centers 15.0 14.0 −23.0 and 20 20 20 boxes; second ligands are box centers 7.0 −3.0 4.0 and 20 20 20 boxes.

## 5. Conclusions

Clinical description of rare cases, such as FPLD3, is highly needed to understand the correlation of clinical and pathological features with their molecular genetic backgrounds. Despite the high efficacy of Pioglitazone in FPLD3 being based on a single case, these findings were supported by structural modeling, molecular docking, and similar findings from previous reports. The findings of this study also improve our approach to early diagnosis of lipoatrophic diabetes and highlight the potential for personalized treatment strategies based on genetic mutations.

## Figures and Tables

**Table 1:** Anthropometric and biochemistry data.

Variables	Before Pioglitazone	After Pioglitazone	Reference Ranges
BMI kg/m^2^	28.5	27.4	
Fasting blood glucose, mg/dL	325	102	<100
HbA1c, %	>14	6.7	<5.7
C-peptide, ng/mL	2.3	1.6	1.1–4.4
Insulin, mIU/L	27	14	0–25
Anti-GAD antibodies, U/mL	<5	<5	<10
Total cholesterol, mg/dL	171	135	<200
HDL cholesterol, mg/dL	22	31	>50
LDL cholesterol, mg/dL	102	74	<130
Triglycerides, mg/dL	>1000	198	<150
Leptin, ng/mL	4.7	2.9	3.7–11.1
Adiponectin, µg/mL	9.3	6.1	8.2–19
ALT, U/L	83	37	<40
AST, U/L	67	26	<40
Creatinine, mg/dL	0.5	0.65	0.6–1.2
Urea, mg/dL	14	12	15–45

## Data Availability

All the necessary information is provided within the manuscript. Any other data that support the findings of this study are available from the first author upon request.

## Supplementary Materials

The following supporting information can be downloaded at: https://www.mdpi.com/article/10.3390/ijms252212060/s1.
