# Supplementary material for: Genomic and Bioinformatics Analysis of Familial Partial Lipodystrophy Type 3 Identified in a Patient with Novel PPARγ Mutation and Robust Response to Pioglitazone
Source: Int J Mol Sci. 2024 Nov 10;25(22):12060. doi: 10.3390/ijms252212060 (PMC11593357; doi:10.3390/ijms252212060)
Supplement: Supplementary file 1 [file ijms-25-12060-s001.zip › ijms-3239096-supplementary.pdf]

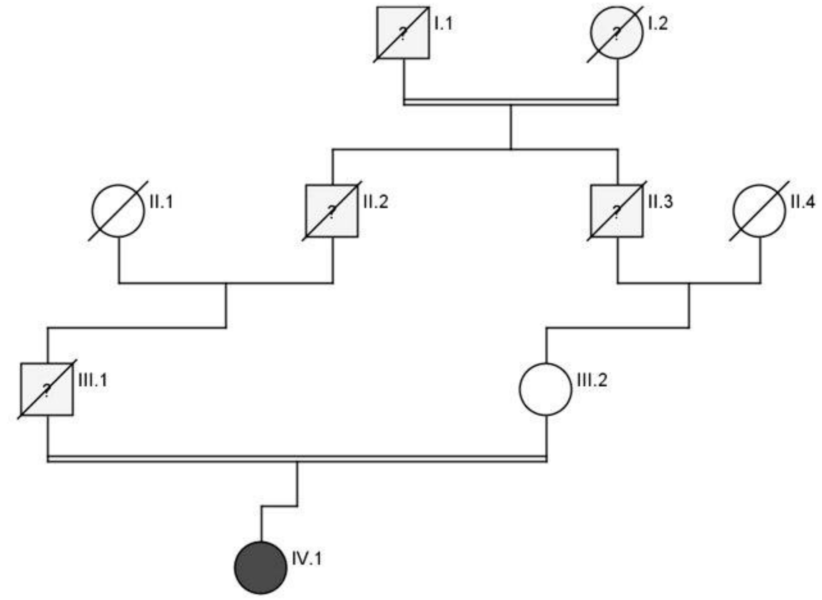

Figure S1. Patient pedigree.

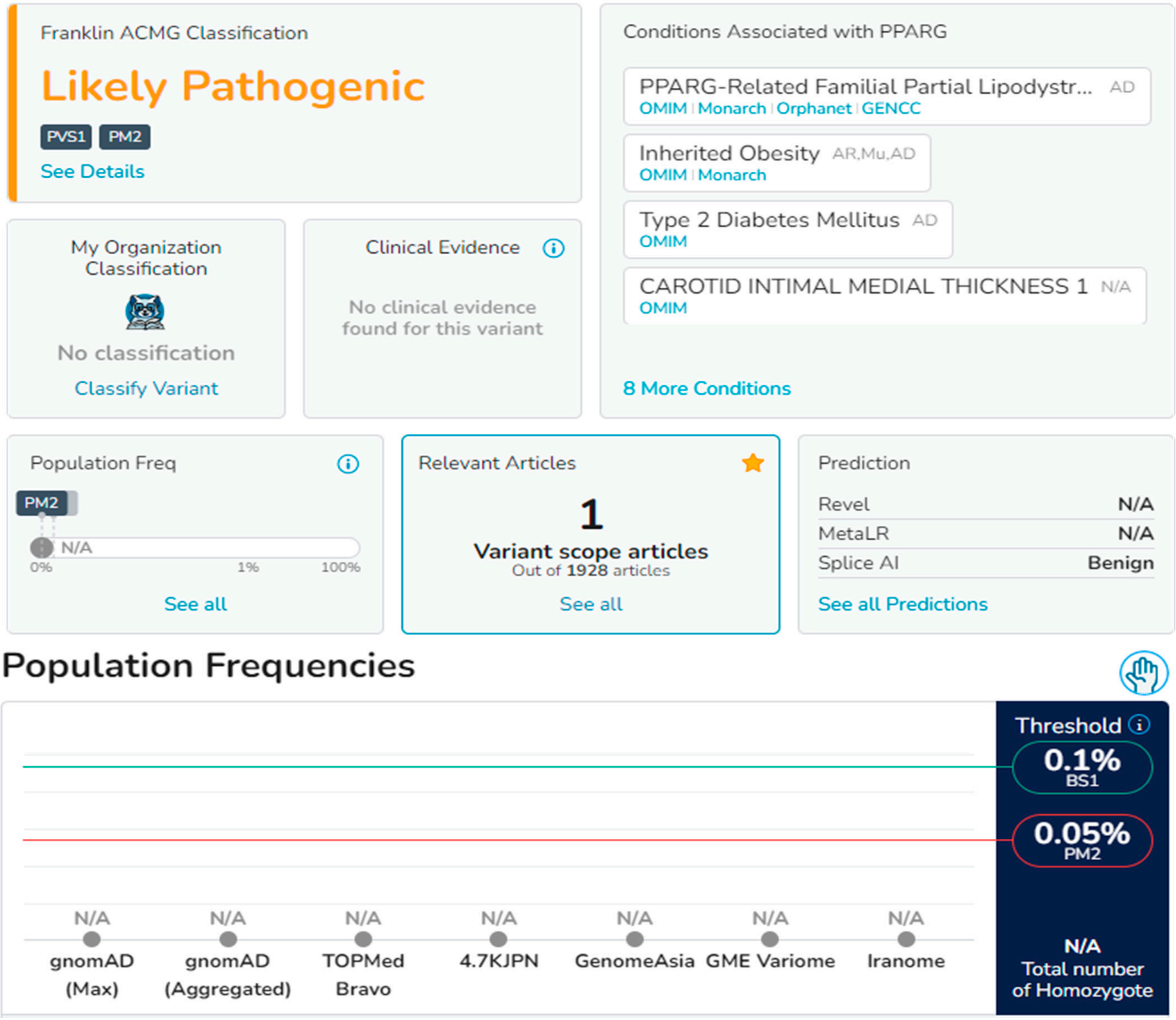

Figure S2. Pathogenicity assessment of the PPARG:c.1024C>T variant using Franklin ACMG Classification, predicting it to be "Likely Pathogenic" under PVS1-PM2 classification.

The Franklin ACMG classification labels the variant as "Likely Pathogenic" based on criteria PVS1 and PM2. This classification aligns with known conditions associated with PPARγ mutations, such as FPLD, inherited obesity, and Type 2 Diabetes Mellitus. However, no clinical evidence has been directly associated with this specific variant, and the mutation has not yet been classified by any specific organization. The figure suggests the need for further investigation, as only one variant-specific article is highlighted from 1,928 available articles. additional models like Revel or MetaLR provide no specific predictions. Finally, the absence of population frequency data for this variant (PVS1-PM2) highlights its rarity, supporting its classification as likely pathogenic in contributing to PPARG-related conditions.

Table S1. Genomic and Molecular Details of the Identified PPARG Mutation.

Gene name	Chromosomal position	Rs ID	Ref/Alt Bases	Effect	HGVS.c	A. F
PPARG	3: 12416908	NA	C>T	Stop gained	c.1024C>T	NA

Table S2. Accession numbers for humans and 10 other primate species from NCBI Website.

Species	Accession Number
Homo sapiens (human)	NM_138711.6
Mus musculus (mouse)	NM_001308354.2
Rattus norvegicus (rat)	NM_001145367.1
Sus scrofa (pig)	NM_214379.1
Canis lupus familiaris (dog)	NM_001024632.2
Macaca mulatta (rhesus monkey)	NM_001032860.1
Oryctolagus cuniculus (rabbit)	NM_001082148.1
Cricetulus griseus (Chinese hamster)	NM_001244281.1
Pan troglodytes	XM_016940489.4
Felis catus	XM_003982496.6
Heterocephalus glaber	XM_004875118.3
